# Impact of continuous nerve block at different iliac fascia compartment regions on postoperative analgesia following total hip arthroplasty: A randomized controlled trial

**DOI:** 10.1097/MD.0000000000044424

**Published:** 2025-09-19

**Authors:** Hong-Chao Zhang, Shou-Fu Wang, Dong Wang

**Affiliations:** a Department of Anesthesiology, Heze Medical College, Heze, China.

**Keywords:** fascia iliaca compartment block, nerve block, pain management, postoperative analgesia, Total hip arthroplasty

## Abstract

**Background:**

This study evaluates the analgesic efficacy of nerve block catheter placement in various regions of the iliac fascia compartment following total hip arthroplasty (THA).

**Methods:**

A total of 90 patients scheduled for unilateral THA were enrolled, comprising 46 males and 44 females, aged 55 to 75 years, with a body mass index of 18.0 to 30.0 kg/m^2^ and American Society of Anesthesiologists classification I or II. Patients were randomized into 3 groups of 30. All groups received general anesthesia in combination with nerve block anesthesia. The nerve block catheter was positioned in distinct regions of the iliac fascia compartment under bedside ultrasound and anteroposterior hip X-ray guidance. Based on anteroposterior hip X-rays, the ilium was divided into 3 regions – medial, middle, and lateral. Catheters were placed in the medial region in group I, the middle region in group II, and the lateral region in group III. The catheter depth within each region was recorded. Postoperative analgesia was managed using nerve block analgesia. The Numerical Rating Scale for pain, muscle strength, sensory level, effective analgesic pump compression counts, and adverse reactions during activity were assessed at 6, 12, 24, 36, and 48 hours postsurgery.

**Results:**

Numerical Rating Scale scores during activity at 12 to 48 hours postoperatively were significantly lower in groups I and II compared with group III (P < 0.05). In addition, the effective compression counts of the analgesic pump were significantly reduced in groups I and II compared with group III (*P* < .05).

**Conclusion:**

Continuous fascia iliaca compartment block provides effective analgesia after THA, with catheter placement in the medial and middle regions offering superior analgesic outcomes compared with the lateral region.

## 1. Introduction

Total hip arthroplasty (THA) is the definitive treatment for advanced hip joint diseases, providing significant pain relief and functional improvement.^[1]^ Despite its benefits, THA is frequently associated with severe postoperative pain, which can impede recovery, delay rehabilitation, and adversely affect long-term outcomes.^[2]^ Inadequately controlled acute pain may escalate into chronic pain, compromising the overall success of the procedure. Thus, effective and safe postoperative analgesia is a critical aspect of THA management.^[3]^

Continuous fascia iliaca compartment block (FICB) has gained attention as a potential strategy for sustained postoperative analgesia in THA.^[4]^ This technique relieves continuous pain, enhances patient comfort, and promotes early mobilization. However, the analgesic efficacy of FICB varies considerably, with previous studies indicating a wide range in the optimal depth of catheter placement within the fascia iliaca compartment, from 5 to 20 cm.^[5]^ These variations suggest that factors such as the depth and location of cannulation significantly influence the analgesic outcomes. Nevertheless, the relationship between the cannulation depth and the specific anatomical area targeted within the iliac fascia remains underexplored.

Lumbar plexus anatomy suggests that nerves are more densely clustered near the midline, indicating that a medially positioned nerve block might provide superior analgesia. This anatomical insight led us to hypothesize that dividing the fascia iliaca compartment longitudinally into medial, middle, and lateral regions relative to the ilium could influence the effectiveness of FICB. Accordingly, our study investigates the impact of catheter placement within these regions on analgesic outcomes, aiming to refine FICB techniques for improved postoperative pain control in THA patients.

## 2. Methods

### 2.1. Study registration and ethical approval

The study was approved by the Ethics Committee of Heze Municipal Hospital (Approval No. 2020-KY002, dated April 11, 2020) and conducted in accordance with the Declaration of Helsinki. Written informed consent was obtained from all participants before enrollment.

The trial was prospectively registered with the China Clinical Trial Registry (ChiCTR2000031784). The original protocol specified a comparison of catheter insertion depths of 5 and 10 cm. However, before enrollment began, the investigators identified a methodological limitation: fixed insertion depth did not reliably correspond to the final position of the catheter tip, owing to interindividual anatomical variability within the fascia iliaca compartment.

To enhance the clinical relevance and precision of the intervention, the protocol was amended to classify patients according to the anatomical region of catheter placement – medial, middle, or lateral – as determined by ultrasound and portable radiography. The revised protocol was approved by the Ethics Committee on April 11, 2020, and all participants provided updated written consent reflecting the modification.

### 2.2. Sample size calculation

Based on preliminary pilot data (N = 5 per group) indicating mean postoperative NRS scores of 1.5 (medial group), 2.2 (middle group), and 2.7 (lateral group) with an estimated SD of 1.1, we performed a power analysis using PASS software (*F*-tests, 1-way ANOVA, fixed effects, omnibus). With an effect size (f) of 0.47, α = 0.05, and power (1−β)=0.95 for 3 groups, the minimum total sample size required was 78 (26 per group). To allow for a 10% attrition rate (e.g., catheter dislodgement, protocol deviations), 90 participants (30 per group) were ultimately enrolled.

### 2.3. Eligibility criteria and group allocation

Patients scheduled to undergo elective unilateral THA at Heze Municipal Hospital between April and August 2020 were screened for eligibility. Inclusion criteria were: age between 50 and 79 years, American Society of Anesthesiologists physical status I or II, and a body mass index of 18 to 30 kg/m². Patients were excluded if they had coagulation disorders; active infection at the puncture site; a history of allergy to local anesthetics; significant hematologic, neurologic, cardiovascular, or cerebrovascular disease; diabetes mellitus or peripheral neuropathy; impaired ability to understand the Numerical Rating Scale (NRS) for pain; use of analgesics within 3 months prior to surgery; participation in another clinical trial within the previous 3 months; or planned revision or bilateral arthroplasty.

Eligible patients were randomly assigned to 1 of 3 groups according to the anatomical region of catheter placement (medial, middle, or lateral) within the fascia iliaca compartment.

### 2.4. Randomization and blinding

Patients were randomly assigned to 1 of 3 groups: Group I, with catheter placement in the medial region of the fascia iliaca compartment; Group II, with catheter placement in the middle region; and Group III, with catheter placement in the lateral region. Randomization was performed using a computer-generated random number table by an independent statistician who was not involved in the clinical study. Allocation concealment was maintained using sealed, opaque, sequentially numbered envelopes. The anesthesiologist responsible for catheter placement was not blinded due to the nature of the intervention; however, the patients, outcome assessors, and data analysts were all blinded to group assignments.

### 2.5. Catheter placement procedure

**Figure 1.**
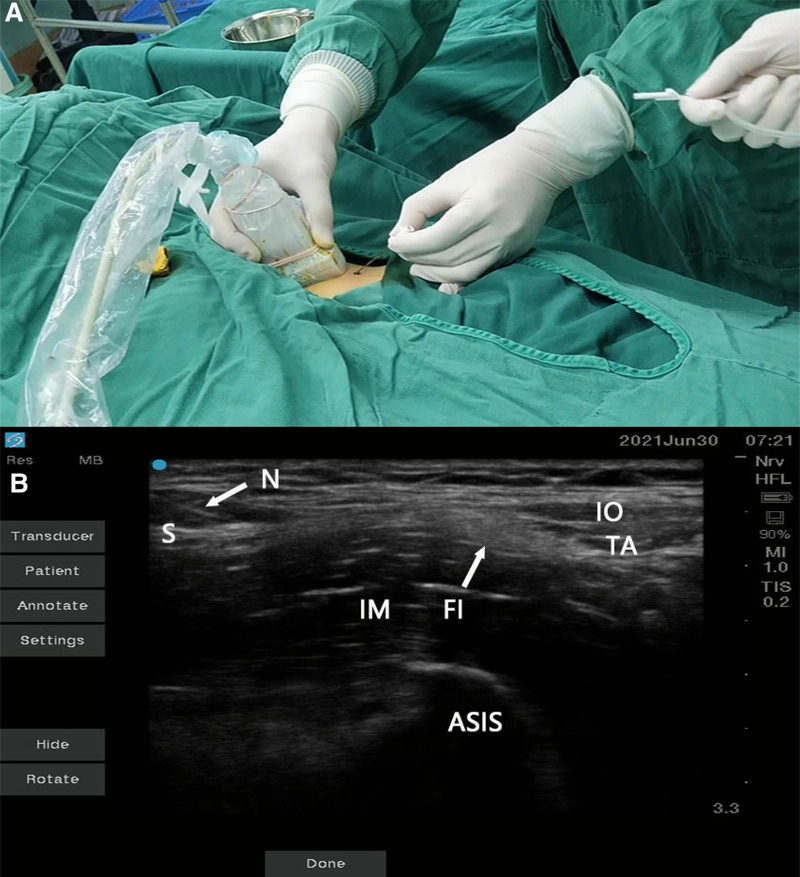
Ultrasound probe placement and imaging for fascia iliaca compartment block. (A) Positioning of the high-frequency ultrasound probe perpendicular to the inguinal ligament over the middle and outer thirds, with the patient in the supine position. (B) Ultrasound image displaying the “hourglass sign,” delineating the IO, TA, the S, and the IM. IM = iliacus muscle, IO = internal oblique, TA = transversus abdominis, S = sartorius muscle.

Patients were positioned supine and a high-frequency probe from a portable ultrasound machine was used to scan the middle and outer thirds of the inguinal ligament, with the probe oriented perpendicular to the ligament (Fig. 1A). The “hourglass sign” was identified (Fig. 1B), delineating the cranial internal oblique muscle, caudal sartorius muscle, and intermediate iliacus muscle. The iliacus fascia, covering the iliacus muscle, was also visualized.

**Figure 2.**
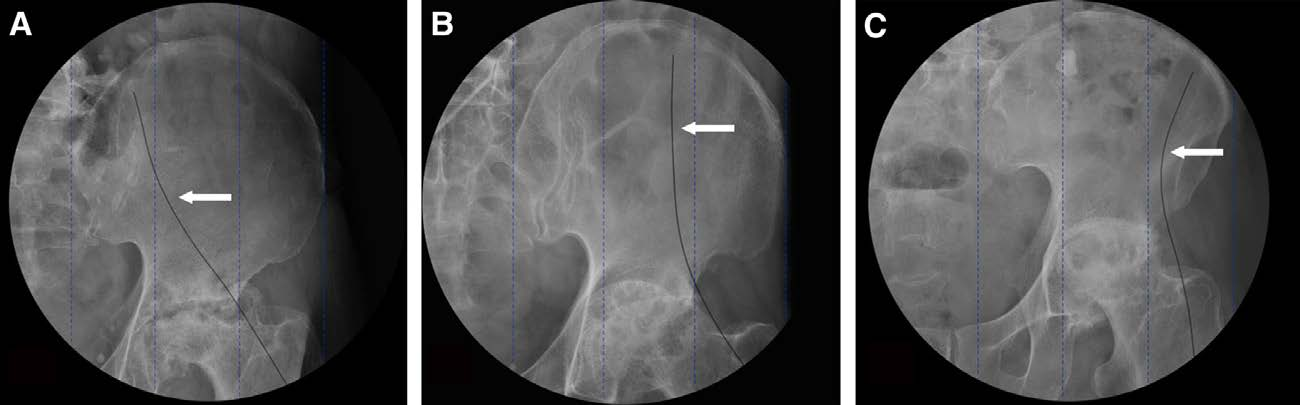
Catheter guidance to target regions within the fascia iliaca compartment using portable X-ray. A portable X-ray machine was utilized to guide the continuous nerve block catheter to the designated target areas within the fascia iliaca compartment: medial region for Group I, middle region for Group II, and lateral region for Group III. The catheter was inserted beneath the deep circumflex iliac artery, and its placement was confirmed to ensure accurate targeting of the fascia iliaca compartment zones.

A single anesthesiologist inserted a continuous nerve block cannula needle into the subiliac fascia beneath the deep circumflex iliac artery. After confirming the absence of blood on aspiration, 30 mL of 0.2% ropivacaine was injected, followed by the insertion of a continuous nerve block catheter. A portable X-ray machine was used to guide the catheter to the designated target area – medial, middle, or lateral region (Fig. 2). The skin insertion depth of the catheter was recorded.

**Figure 3.**
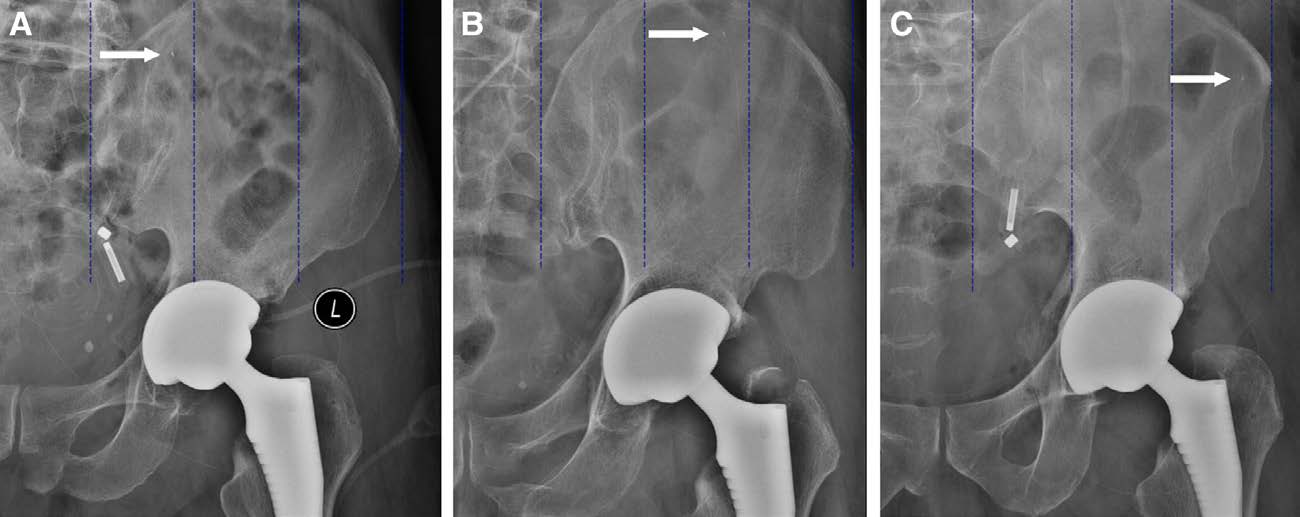
Postoperative X-ray confirmation of catheter placement. Postoperative X-ray images showing the catheter placement relative to the preoperative position. The black arrow indicates the tip of the catheter, confirming that there was no displacement within the fascia iliaca compartment. This verification ensured consistent analgesic delivery throughout the postoperative period.

A catheter was then threaded through the needle and advanced to the assigned target region – medial, middle, or lateral – within the fascia iliaca compartment. Final positioning was confirmed by portable radiography (Fig. 3). The insertion depth was recorded, and the catheter was secured.

### 2.6. Anesthesia protocol

All patients were routinely monitored for heart rate, blood pressure, electrocardiogram (ECG), and oxygen saturation (SpO_2_). Following catheter insertion, general anesthesia was induced with midazolam (0.05 mg/kg), sufentanil (0.5 µg/kg), propofol (1.5 mg/kg), and cisatracurium (0.15 mg/kg). After confirming loss of consciousness and satisfactory muscle relaxation, endotracheal intubation was performed. Mechanical ventilation was initiated (volume-controlled ventilation, tidal volume 8 ml/kg, ventilation frequency 13 breaths/min, and inspiration-expiration ratio 1:2). Anesthesia was maintained with sevoflurane (1.0%–2.0%), propofol (2–4 mg·kg⁻¹·h⁻¹), and remifentanil (0.10–0.15 µg·kg⁻¹·min⁻¹). The BIS value was maintained between 40 and 60, and end-tidal CO₂ (PetCO_2_) was maintained between 35 and 45 mm Hg. Sevoflurane was discontinued 40 minutes before the end of the surgery, and propofol and remifentanil were stopped 5 minutes before surgery completion. Postoperatively, the nerve block catheter was connected to an analgesic pump delivering 0.2% ropivacaine (300 ml) with a background infusion rate of 5 ml/h, a bolus dose of 5 ml, and a lockout interval of 30 minutes. According to the Department of Orthopaedics clinical pathway, patients in all 3 groups received controlled medication 10 minutes before functional exercise. If the NRS score exceeded 4, 30 mg of ketorolac tromethamine was administered intravenously. If pain persisted after 30 minutes (NRS score > 4), 30 mg of pentazocine was given intramuscularly.

### 2.7. Outcome measures

NRS scores during activity were recorded at 6, 12, 24, 36, and 48 hours postoperatively. The number of effective analgesic pump compressions, cumulative ropivacaine dosage, and rescue analgesia with ketorolac tromethamine were also recorded. Sensory assessments of light touch and temperature were conducted on the skin of the affected side (including the femoral nerve, lateral femoral cutaneous nerve, iliohypogastric nerve, and obturator nerve) and the contralateral side at 6, 12, 24, 36, and 48 hours postoperatively. Sensory loss was scored as follows: no loss (0 points), loss of temperature sensation (1 point), and loss of both temperature sensation and light touch (2 points). A nerve block was considered effective if either touch or temperature sensation was impaired. After catheter removal at 48 hours postoperatively, bacterial cultures were performed, and adverse reactions were recorded.

### 2.8. Statistical analysis

Data analysis was performed using SPSS 20.0 (Chicago). Normally distributed continuous variables were expressed as mean ± standard deviation and compared using a 1-way analysis of variance. Non-normally distributed data were expressed as median (M) and interquartile range (IQR) and compared using the *t*-test. Categorical variables were expressed as counts (percentages) and analyzed using Fisher exact test. A *P*-value of <.05 was considered statistically significant.

## 3. Results

A total of 90 patients were enrolled in this study, and all participants completed the trial. There were no statistically significant differences among the 3 groups in terms of gender, age, American Society of Anesthesiologists classification, body mass index, or operative time (Table 1). In Group I, catheters were placed in the medial region with a mean depth of 16.2 ± 1.1 cm. In Group II, catheters were placed in the middle region with a mean depth of 10.3 ± 1.2 cm. In Group III, catheters were placed in the lateral region with a mean depth of 6.7 ± 0.7 cm. The catheter depths differed significantly among the 3 groups (*P* < .05).

**Table 1:** General characteristics of the participants.

Items	Group I(n = 30)	Group II(n = 30)	Group III(n = 30)	*P*
Age, year	65.1 ± 6.5	63.5 ± 5.8	64.1 ± 6.0	n.s.
Sex,male/female	13/17	17/13	16/14	n.s.
Body mass index, kg/m^2^	26.2 ± 5.3	27.1 ± 5.4	26.5 ± 5.3	n.s.
Asa grade	1.5 ± 0.5	1.5 ± 0.4	1.5 ± 0.7	n.s.
Operation time,min	142.2 ± 11.5	138.1 ± 13.6	137.4 ± 13.9	n.s.

ASA = American Society of Anesthesiologists, n.s. = not significant.

At 6 hours postoperatively, there were no statistically significant differences in NRS scores during activity among patients in Groups I, II, and III (*P* > .05). However, at 12, 24, 36, and 48 hours postoperatively, there were no significant differences in NRS scores between Groups I and II (*P* > .05). At the same time, patients in Group III had significantly higher NRS scores than those in Groups I and II (*P* < .05) (Table 2).

**Table 2:** Comparison of NRS scores between the 3 groups at postoperative time points

Time	6 h	12 h	24 h	36 h	48 h
Group I	1.8 ± 0.6	2.2 ± 0.7*	2.9 ± 0.7*	2.1 ± 0.8*	1.6 ± 0.4*
Group II	1.8 ± 0.7	2.4 ± 0.5*	3.0 ± 0.8*	2.3 ± 0.6*	1.7 ± 0.5*
Group III	1.9 ± 0.6	3.3 ± 0.9	3.7 ± 0.5	3.1 ± 0.7	2.6 ± 0.7

* *P *< .05 considered statistically significant compared with Group III.

Regarding sensory block effects, there were no significant differences in obturator nerve, femoral nerve, or lateral femoral cutaneous nerve block scores among the 3 groups at 6 hours postoperatively. From 12 to 48 hours postoperatively, there were no significant differences in obturator nerve and femoral nerve block scores between Groups I and II (*P* > .05). However, both were significantly higher than in Group III (*P* < .05). There were no significant differences in lateral femoral cutaneous nerve block scores among the 3 groups (*P* > .05) (Table 3).

**Table 3:** Comparison of nerve block in 3 groups at each time point after operation.

	Group	6 h	12 h	24 h	36 h	48 h
Obturator nerve	Group I	1.7 ± 0.4	1.4 ± 0.5*	1.1 ± 0.3*	1.2 ± 0.4*	1.2 ± 0.4*
	Group II	1.7 ± 0.4	1.4 ± 0.4*	1.1 ± 0.3*	1.1 ± 0.3*	1.0 ± 0.1*
	Group III	1.6 ± 0.4	1.0 ± 0.4	0.7 ± 0.4	0.6 ± 0.4	0.6 ± 0.4
Femoral nerve	Group I	1.7 ± 0.4	1.4 ± 0.5*	1.1 ± 0.3*	1.2 ± 0.4*	1.1 ± 0.3*
	Group II	1.8 ± 0.3	1.2 ± 0.4*	1.1 ± 0.3*	1.2 ± 0.4*	1.1 ± 0.3*
	Group III	1.6 ± 0.4	0.8 ± 0.4	0.8 ± 0.4	0.8 ± 0.3	0.7 ± 0.4
Nervus cutaneus femoris lateralis	Group I	1.6 ± 0.4	1.2 ± 0.4	1.2 ± 0.4	1.1 ± 0.3	1.1 ± 0.3
	Group II	1.6 ± 0.4	1.1 ± 0.3	1.3 ± 0.4	1.1 ± 0.3	1.1 ± 0.3
	Group III	1.6 ± 0.4	1.0 ± 0.2	1.4 ± 0.5	1.2 ± 0.4	1.2 ± 0.4

* *P *< .05 considered statistically significant compared with Group III.

The number of effective compressions of the analgesic pump was significantly lower in Groups I and II compared to Group III (*P* < .05). There were no significant differences between Groups I and II in terms of cumulative ropivacaine dosage and the number of rescue analgesia administrations with ketorolac tromethamine (*P* > .05); however, both were significantly lower than in Group III (*P* < .05). The 3 groups had no significant difference in the time to first ambulation (Table 4).

**Table 4:** Comparison of other analgesic effect indexes among 3 groups.

	Effective pressing times of analgesic pump	Total times of analgesic pump pressing	Dosage of ropivacaine	Remedial analgesia	First time out of bed (h)
Group I	4.3 ± 2.2*	6.5 ± 3.4*	230.8 ± 3.7*	0.6 ± 0.7*	24.3 ± 1.7
Group II	6.7 ± 3.0*	10.4 ± 2.6*	231.3 ± 3.1*	0.7 ± 0.5*	26.1 ± 2.0
Group III	8.5 ± 3.2	15.3 ± 3.7	257.0 ± 3.8	1.1 ± 0.6	26.7 ± 2.2

* *P *< .05 considered statistically significant compared with Group III.

Adverse events observed during the study included nausea, vomiting, drowsiness, and transient sensory changes. No cases of local anesthetic systemic toxicity, motor deficits, catheter-site infection, or falls were reported in any group. Bacterial cultures obtained from catheter tips 48 hours after removal were negative in all patients. There were no statistically significant differences in the incidence of adverse events among the 3 groups (Table 5).

**Table 5:** Comparison of postoperative adverse reactions among 3 groups (percentage).

Group	Nausea	Vomiting	Lethargy
Group I	2 (6.7)	1 (3.3)	1 (3.3)
Group I	2 (6.7)	1 (3.3)	0 (0)
Group I	3 (10)	1 (3.3)	1 (3.3)

## 4. Discussion

Postoperative pain following THA predominantly includes incision pain and pain during functional exercises.^[6]^ Persistent severe pain not only delays recovery but also heightens the risk of complications such as gastrointestinal dysfunction, decreased pulmonary ventilation, pulmonary embolism, and deep vein thrombosis.^[7]^ Effective and safe analgesia is, therefore, critical to alleviating pain, reducing postoperative complications, and enhancing the quality of life for patients undergoing THA. In this study, we demonstrated that continuous FICB provides effective postoperative analgesia, with catheterization in the medial and middle zones of the fascia iliaca compartment yielding superior analgesic effects compared to the lateral zone.

FICB has effectively blocked the obturator nerve, lateral femoral cutaneous nerve, and femoral nerve, making it a viable strategy for postoperative analgesia after hip replacement. Previous studies using MRI have indicated that the origins of the nerves innervating the hip joint are located near the posterior iliac fascia, dispersing downward and outward.^[8]^ The proximity of the FICB above the inguinal ligament to the lumbar plexus origin suggests a potentially broader drug diffusion and superior analgesic effect compared to FICB below the inguinal ligament.^[9]^ Clinically, we observed that catheterization in the fascia iliaca space above the inguinal ligament could reach depths up to 25 cm, with angiographic evidence supporting the diffusion of the anesthetic solution to the paravertebral space at such depths.^[10]^ This suggests that the blocking effect and range of FICB might vary based on the specific catheterization site.

However, there currently needs to be standardized depth for catheter placement, and a need for large-scale retrospective studies limits the establishment of definitive guidelines. In this study, we employed ultrasound combined with a portable C-arm to ensure accurate catheter placement centrally within the designated zones, thereby reducing the risk of error from catheterization near the borders of adjacent areas. The nerve block catheter was inserted below the deep circumflex iliac artery and guided to the target position with ultrasound and bedside hip anteroposterior X-ray. A follow-up X-ray was conducted before catheter removal to confirm that the catheter had not shifted, ensuring consistency in the results.

Our findings indicated no significant differences in NRS scores among the 3 groups within the first 6 hours postoperatively. This is likely attributable to the initial administration of 30 mL of 0.2% ropivacaine, which provides analgesia for approximately 8 hours. However, from 12 to 48 hours postoperatively, NRS scores were consistently lower in the medial and middle zones compared to the lateral zone. This may be due to the anatomical proximity of the catheterization sites in the medial and middle zones to the lumbar plexus, resulting in more effective analgesia than the lateral zone. Some studies have suggested that the fascia iliaca compartment features both an upper and lower opening, allowing local anesthetic injected into the space to spread through the upper opening to block the lumbar plexus nerves, thereby producing a broader and more effective nerve block.^[11,12]^ The similar analgesic effects observed in the medial and middle zones may be due to both catheter placements near the upper opening, allowing a similar amount of local anesthetic to reach the lumbar plexus.^[13]^

In contrast, catheter placement in the lateral zone may be farther from the upper opening, limiting the diffusion of the anesthetic to the lumbar plexus and thereby reducing the effectiveness of the nerve block. Postoperatively, sensory testing across different time points showed no significant differences in the block effects of the obturator, femoral, and lateral femoral cutaneous nerves at 6 hours. This may be due to the initial local anesthetic dose and the prolonged action of ropivacaine. However, from 12 to 48 hours postoperatively, the obturator and femoral nerve block effects were superior in the medial and middle zones compared to the lateral zone, likely due to the catheter tips being closer to these nerves in the iliac fascia space. Conversely, the lateral femoral cutaneous nerve block showed no significant differences among the 3 groups, possibly because the catheter tip in the lateral zone was closer to this nerve. In contrast, the medial and middle zone catheters affected it via the upper opening of the fascia iliaca space.

Postoperative adverse reactions, such as nausea and vomiting, were observed across all groups, likely due to the effects of general anesthesia and the physiological stress of surgery, with no significant differences in incidence. Bacterial cultures performed 48 hours after catheter removal were negative in all cases, indicating that catheter removal at this time is safe.^[14]^

Despite the valuable insights provided by our study on the efficacy of continuous FICB for postoperative analgesia in THA, several limitations must be acknowledged. The relatively small sample size and single-center design may limit the generalizability of our findings, highlighting the need for more extensive multicenter studies to validate these results. Additionally, while ultrasound and X-ray guidance were employed to ensure precise catheter placement, potential variability in the exact catheter tip location could affect the consistency of the nerve block, suggesting that further refinement in placement techniques or advanced imaging methods may be beneficial. The study also did not explore the impact of different doses, concentrations, or types of local anesthetics on block efficacy, nor did it assess long-term outcomes such as chronic pain development or recovery beyond the immediate postoperative period. Moreover, the focus on short-term outcomes, with follow-up limited to 48 hours, precludes a comprehensive understanding of the sustained efficacy, potential complications, and overall patient satisfaction over a more extended period. Future research should address these limitations by incorporating extended follow-up periods and broader assessments to optimize postoperative analgesia strategies and enhance patient outcomes after THA.

## 5. Conclusion

Our study provides evidence that placing a continuous FICB catheter in the medial and middle zones of the fascia iliaca compartment offers superior postoperative analgesia compared to placement in the lateral zone. Future studies should focus on establishing standardized guidelines for catheter placement depth and explore the long-term outcomes of different catheterization strategies. Additionally, the impact of varying doses and concentrations of local anesthetics on the efficacy of FICB warrants further investigation. These efforts could improve pain management protocols and enhance patient outcomes following THA.

## Author contributions

**Conceptualization:** Hong-Chao Zhang, Dong Wang.

**Data curation:** Hong-Chao Zhang, Shou-Fu Wang, Dong Wang.

**Formal analysis:** Hong-Chao Zhang, Shou-Fu Wang, Dong Wang.

**Funding acquisition:** Hong-Chao Zhang, Dong Wang.

**Investigation:** Hong-Chao Zhang, Shou-Fu Wang, Dong Wang.

**Methodology:** Hong-Chao Zhang, Shou-Fu Wang, Dong Wang.

**Project administration:** Hong-Chao Zhang, Shou-Fu Wang, Dong Wang.

**Resources:** Hong-Chao Zhang, Shou-Fu Wang, Dong Wang.

**Software:** Hong-Chao Zhang, Shou-Fu Wang, Dong Wang.

**Supervision:** Hong-Chao Zhang, Shou-Fu Wang, Dong Wang.

**Validation:** Hong-Chao Zhang, Dong Wang.

**Visualization:** Hong-Chao Zhang, Dong Wang.

**Writing** – **original draft:** Hong-Chao Zhang, Dong Wang.

**Writing** – **review & editing:** Hong-Chao Zhang, Dong Wang.
